# Effect of warm-up and muscle fatiguing exercise on knee joint sounds in motion by vibroarthrography: A randomized crossover trial

**DOI:** 10.1371/journal.pone.0257652

**Published:** 2021-09-17

**Authors:** Sarah Tenberg, Kristin Kalo, Daniel Niederer, Lutz Vogt

**Affiliations:** 1 Department of Computer Science / Therapy Sciences, University of Applied Sciences Trier, Trier, Germany; 2 Department of Sports Medicine, Disease Prevention and Rehabilitation, Institute of Sport Science, Johannes Gutenberg University Mainz, Mainz, Germany; 3 Institute of Occupational, Social and Environmental Medicine, University Medical Center of the University of Mainz, Mainz, Germany; 4 Department of Sports Medicine and Exercise Physiology, Goethe University Frankfurt am Main, Frankfurt, Germany; Universita degli Studi di Milano, ITALY

## Abstract

Vibroarthrography measures joint sounds caused by sliding of the joint surfaces over each other. and can be affected by joint health, load and type of movement. Since both warm-up and muscle fatigue lead to local changes in the knee joint (e.g., temperature increase, lubrication of the joint, muscle activation), these may impact knee joint sounds. Therefore, this study investigates the effects of warm-up and muscle fatiguing exercise on knee joint sounds during an activity of daily living. Seventeen healthy, physically active volunteers (25.7 ± 2 years, 7 males) performed a control and an intervention session with a wash-out phase of one week. The control session consisted of sitting on a chair, while the intervention session contained a warm-up (walking on a treadmill) followed by a fatiguing exercise (modified sit-to-stand) protocol. Knee sounds were recorded by vibroarthrography (at the medial tibia plateau and at the patella) at three time points in each session during a sit-to-stand movement. The primary outcome was the mean signal amplitude (MSA, dB). Differences between sessions were determined by repeated measures ANOVA with intra-individual pre-post differences for the warm-up and for the muscle fatigue effect. We found a significant difference for MSA at the medial tibia plateau (intervention: mean 1.51 dB, standard deviation 2.51 dB; control: mean -1.28 dB, SD 2.61 dB; F = 9.5; p = .007; η^2^ = .37) during extension (from sit to stand) after the warm-up. There was no significant difference for any parameter after the muscle fatiguing exercise (p > .05). The increase in MSA may mostly be explained by an increase in internal knee load and joint friction. However, neuromuscular changes may also have played a role. It appears that the muscle fatiguing exercise has no impact on knee joint sounds in young, active, symptom-free participants during sit to stand.

## Introduction

Vibroarthrography is a non-invasive method for the assessment of knee joint sounds [1]. During active movements, knee acoustic emissions and vibrations are generated by the articulation of the joint components and the sliding of the joint surfaces against each other [2, 3].

Current literature has shown that various activities of daily living and standardized extension-flexion cycles with different (external) loads lead to variations in the joint sound frequency and amplitude of symptom-free knees [4–6]. When potential factors for influencing the acoustic signal of the knee (e.g., knee angular velocity, age, etc.) are controlled, knee joint sounds can reflect knee joint moments and, thus, knee loads [7]. Different knee joint sounds are produced by different movement conditions (open versus closed kinetic chain) [5]. Previous findings suggest that the knee joint sounds can indicate biomechanical changes of the joint. To date, it has not been investigated whether the acute effects of physical activity on the joint can be measured by joint sounds. The physiological mechanisms that could influence the knee joint sound include, in particular, the distribution of pressure on the cartilage and the concentration of the synovial fluid [8, 9]. According to Kersting et al. [10], muscular co-activation has the greatest influence on cartilage volume changes, thus, muscle activation may be of great importance [5, 11].

Preexercise warm-ups are widely recommended in sports practice to achieve peak performance and prevent injuries [12, 13]. Such warm-ups have an effect on the lubrication and stabilization of the joint [14–17]. The preexercise movement leads to a short-term accumulation of serum hyaluronic acid [14], which can then be absorbed by the surrounding structures such as the joint. Furthermore, the temperature elevation leads to lubrication of the viscolelastic substances, such as hyaluronic acid, in the synovial fluid [15]. Hyaluronic acid facilitates the sliding of the joint surfaces against each other [16] and, thus, ensures a friction-reduced movement of the joint. Warm-ups further improve the neuromuscular reaction speed [17], which, therefore, lead to better stabilization of the joint. Consequently, a better joint lubrication and joint stability result in reduced joint friction and a better arthrokinematic of the joint. Hence, a warm up may be considered as bio-positive stimulus for the joint.

In contrast to that, exercise-induced fatigue can lead to an increased joint load and a higher injury risk of the joint [18, 19] and, thus, it may be considered as a bio-negative stimulus for the joint. Muscle fatigue may impair the arthrokinematic motion quality of the knee joint due to the impact on muscular or muscle-articular stiffness as well as neuromuscular control [20–23]; this can lead to reduced joint stability [24]. Furthermore, a high load with increased pressure on the joint can lead to increased viscosity of the synovial fluid [25] and, consequently, reduce the sliding ability of the joint surfaces [16]. Although not investigated as yet, exercise-induced changes may also impact on the knee joint sounds.

If local changes of physical activity such as a warm up or a muscle fatiguing exercise would alterate the knee joint sounds, a non-invasive and feasible assessment tool, for example, vibroarthrography, would be of great relevance to monitor a healthy, friction-free movement and/or wear of the joint. This is a particularly significant issue because excessive joint friction has been linked to articular cartilage degradation and may contribute to premature wear and knee pathologies (e.g., chondromalacia or osteoarthritis) [26].

The present study, therefore, examined the effects of a warm-up and a muscle fatiguing exercise on knee joint sounds which were assessed by vibroarthrography during the sit-to-stand movement. We hypothesized that a general warm-up reduces the knee sound amplitude, in comparison to a control measurement, by improving lubrication [14, 16] as well as elasticity of articular cartilage [9] and by improving neuromuscular control. Furthermore, we hypothesize that the execution of a knee-loading exercise until task failure increases the sound amplitude, in comparison to a control measurement, by reducing neuromuscular control and joint stability [20–24] as well as increasing the viscosity of the synovial fluid and, thus, reducing the smooth gliding of the joint surfaces [16, 25].

## Materials and methods

### Study design and ethics

A randomized, controlled cross-over design was adopted. The study was conducted according to the ethical guidelines of the Helsinki Declaration (with its recent modification of Fortaleza, 2013) and was approved by the local ethics committee of the Department of Psychology and Sports Sciences, Goethe University Frankfurt am Main. Prior to participation in the study, all participants provided written informed consent.

### Participants

Nineteen (19) healthy adults (mean 25.6 years old, standard deviation 2.0 years, 8 males) were recruited by means of personal addressing. Exclusion criteria comprised a history of knee injuries, acute or incomplete healed injuries or operations, current knee pain/muscle soreness, other diseases that affect walking ability/ standing stability and intense physical activity in the last 48 hours. In addition, the participants were asked if muscle soreness was present before each measurement day and they were asked to visit the test station under the same conditions (by bus, car, bicycle or walking).

### Experimental protocol

**Fig 1:**
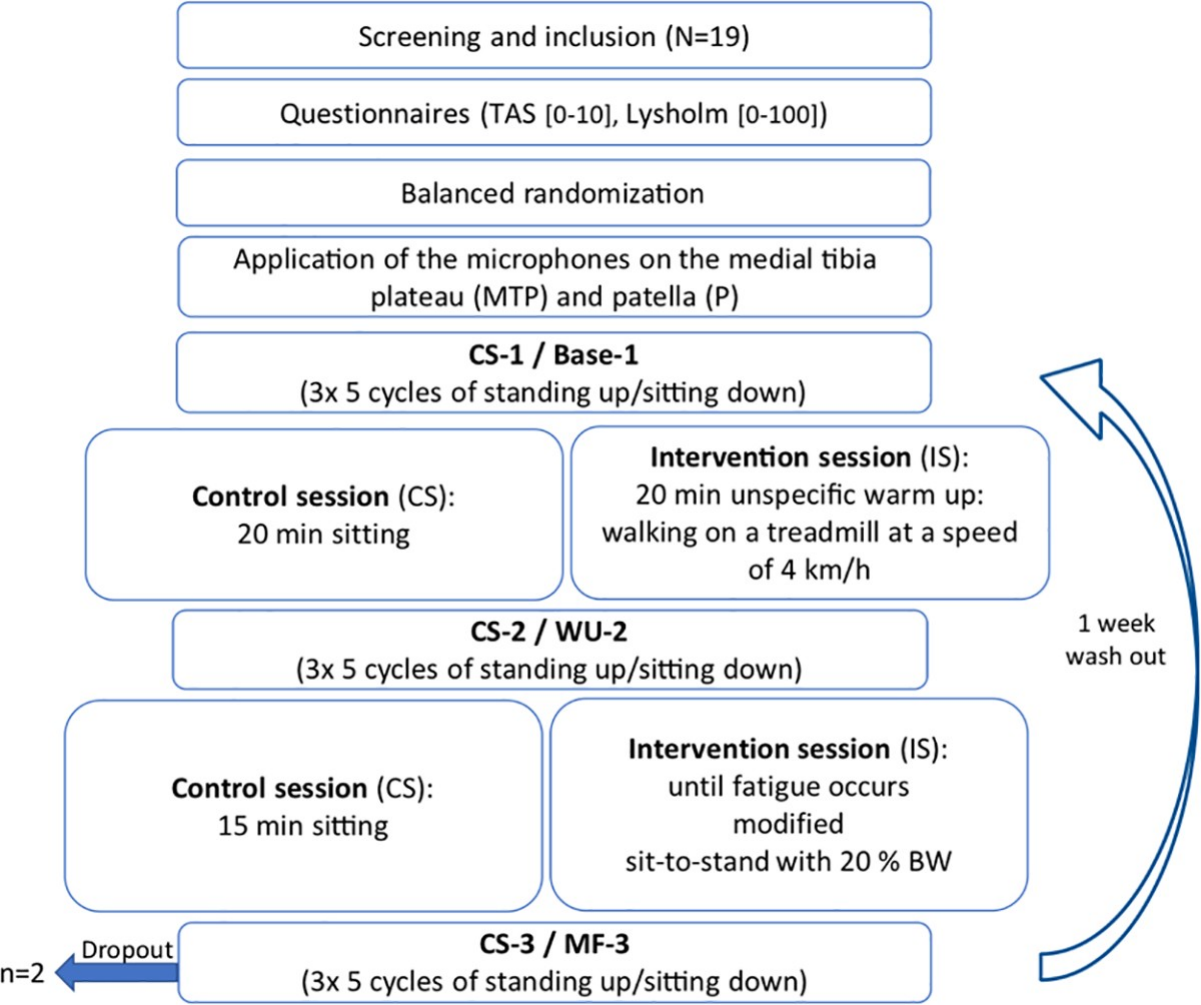
Study protocol. WU: warm up; MF: muscle fatigue; TAS: Tegner Activity Scale; BW: body weight.

Each enrolled participant completed two sessions on two separate days. A wash-out phase of seven days separated these two sessions. The sequence of the session was randomized and counterbalanced. The control session consisted of “do-nothing” while the intervention session contained the warm-up and muscle fatiguing exercise protocols. The two sessions were executed at the same time of day. A schematic description of the study protocol is shown in Fig 1.

After screening for inclusion and exclusion criteria, demographic and sport specific questionnaires were completed. Before and after the warm-up, as well as after the muscle fatiguing, knee joint sounds were recorded during sit-to-stand movements. In the control session, knee joint sounds were measured equivalently (at the same time points) to the intervention session, but instead of the warm up and fatiguing exercise, participants rested on a chair.

### Interventions

#### Warm-up

The unspecific warm-up consisted of 20 minutes of walking on a treadmill (quasar med; h/p/cosmos sports & medical gmbh; Nussdorf-Taunstein, Germany) at a speed of 4 km/h. The Borg rating of perceived exertion (RPE scale 6–20) of the participants was queried before starting the measurement and after the warm-up.

#### Fatiguing exercise

**Fig 2:**
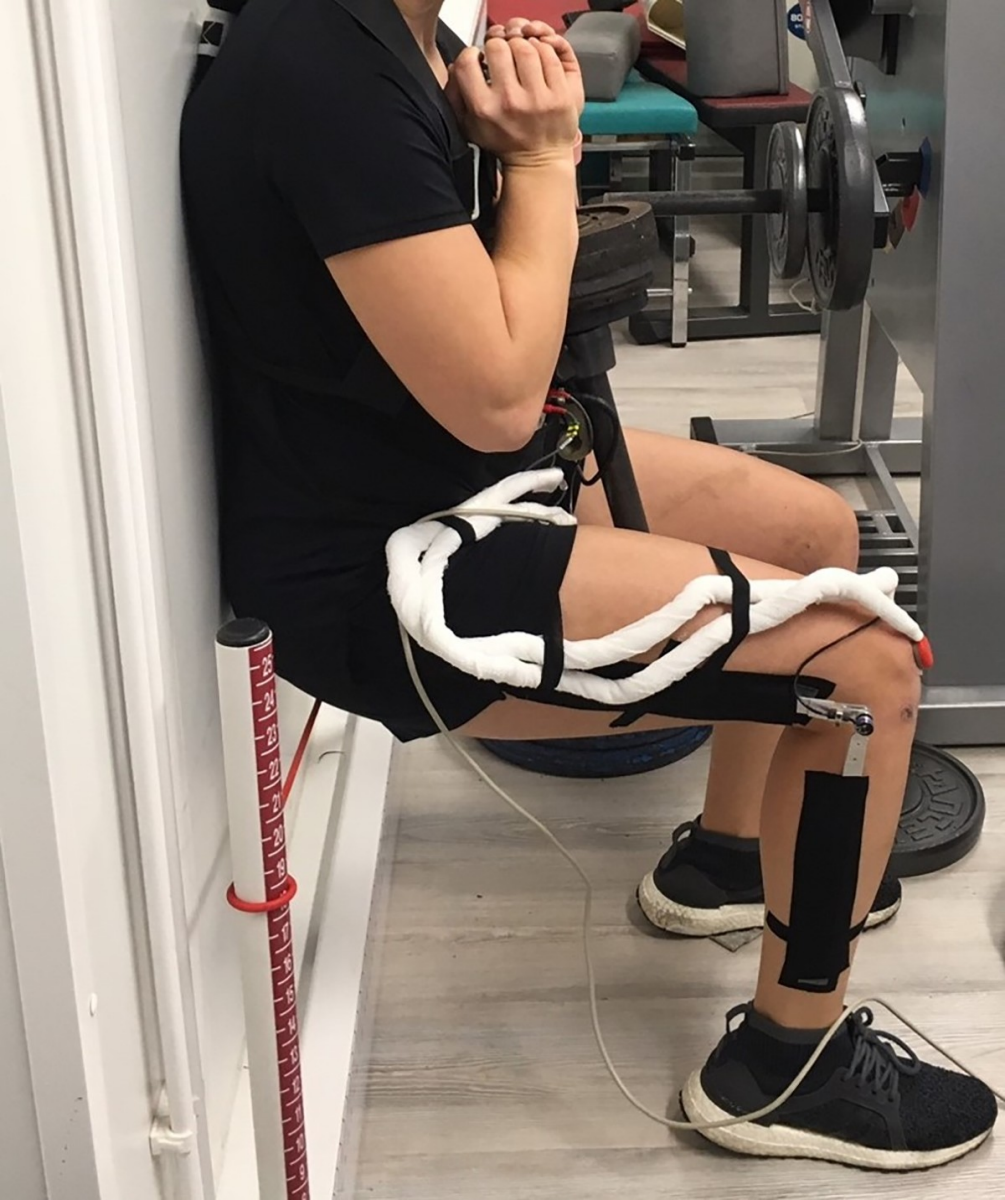
Sitting position using the tactile stimulus from a tensioned rope during the modified sit-to-stand movement and with an additional weight equal to 20% of the participant’s body weight.

A sit-to-stand task for muscle fatigue [27] was conducted where the participants were subjected to an extra weight of 20% of their own body weight (Fig 2). The movement was modified, so that the participants were leaning with their back against a wall. The feet were placed in front of the body so that the knee joint was in 90° flexion during the sitting position. Upon reaching 90° knee flexion, the participants received a tactile stimulus from a tensioned rope that was adjusted to the height of the lower leg. The pilot measurements showed that the upper body should be fixed to the wall in order to reduce the activation of the trunk muscles, thus ensure that the anterior and posterior thigh muscle chains acting on the knee joint were primarily stressed [28]. The tactile stimulus was chosen, instead of sitting down on a chair, in order to generate sustained muscle activity to making certain a task failure due to exhaustion of the athletic study population.

The participants moved from the sitting to a standing position and vice versa at a frequency of 30 repetitions per minute, indicated by using a metronome. Task failure was deemed reached as soon as the frequency could not be maintained (4 cycles behind) or when the movement became uncontrolled, thus, the participants were considered to be fatigued. After the exercise, Borg rating of perceived exertion (RPE 6–20) of the participants was recorded. Time until exhaustion was noted.

### Outcomes and measurements

#### Vibroarthrography recordings

For the vibroarthrographic recording, an activity of daily living, the sit-to-stand movement on a height-adjustable bench, was performed. Each cycle commenced in the sitting position with the knee, ankle and hip angle at 90°, followed by standing up to an upright standing position (knee angle of 180°) and was completed by sitting down again (knee angle returning to 90°). The movements had to be performed fluently at a speed of 2 seconds for standing up and 2 seconds for sitting down. Pacing was controlled by using a visual metronome. Three sets of five repetitions were performed. The break between the sets was 10 seconds.

Knee sounds were recorded with a sampling rate of 16,000 Hz by means of two acoustic sensors with a diameter of 3 cm (microphones; Knowles Electronics, LLC. Itasca, IL, USA). The microphones were placed over the medial tibia plateau (with the upper limit of the sensor 2 cm below the joint space) and over the center of the patella [29]. These anatomical positions ensure the best contact area and are closest to the bone, and, therefore, reduce the influence of skin and connective tissue on knee sounds as much as possible [1, 30]. The microphones were attached to the skin using double-sided tape, whilst adhesive tape was used for the wires. Prior to the placement, the skin was prepared by hair removal and alcohol cleaning. The examined leg was randomly selected.

By using the same electrode placement and measurement protocol, this method was shown to possess an excellent intra-session reliability for the medial tibia plateau (ICC from .85 to .95; CV ≤ .06) and a good intra-session reliability for the patella (ICC from .73 to .87; CV ≤ .07). However, it yielded inconsistent results for the interday reliability at the medial tibia plateau and patella (ICC from 0 to .82) [29], therefore, the electrodes were not moved during the entire session (including warm-up and muscle fatigue periods). In addition, a synchronized electrogoniometer (Penny and Giles Biometrics, Newport, UK), was attached laterally to the leg with the rotation axis aligned to the knee joint.

### Outcomes and data processing

#### Vibroarthrography

The primary outcome was the mean signal amplitude (dB, MSA), while the secondary outcome was the median power frequency (Hz, MDF).

The knee joint sound signals were digitized using an A/D converter (National Instruments Corp., Austin, TX, USA). The converter was placed in a custom-made framework around the waist of the participant. The first set of five repetitions was always evaluated. If errors during the execution of the movement or technical problems occurred, the 2^nd^ or 3^rd^ set was used. The signals were processed with a digital bandpass filter (100–300 Hz). The individual movement cycles (extension: from sit-to-stand; flexion: from stand-tosit) were extracted by means of the knee angle of the electrogoniometer. Subsequently, the data were sampled down to 1,000 Hz [31] and the signal amplitude was rectified. The mean and median of five cycles were calculated for the extension and flexion movements of the knee using Matlab (version R2018b, MathWorks, Natick, MA, USA). The data preparation process can be found in detail in Kalo et al. [6].

#### Tegner Activity Scale

The Tegner Activity Scale is used to assess the activity level from daily living to high level competitive sport [32]. The scale ranges from 0 to 10, with values above 6 only being achieved by people who participate in recreational or competitive sports [32]. The Tegner Activity Scale demonstrates good test-retest reliability (ICC .82) and is a valid measurement tool [33, 34].

### Statistics

The statistical analysis was conducted with SPSS version 25 (SPSS Inc., Chicago, USA) and Microsoft Excel 2013 for Windows (Microsoft Corporation, Redmond, USA). The level of significance was set, a priori, to p < .05 for all tests.

The data was checked for underlying assumptions for calculating parametrical statistics (normal distribution, variance homogeneity, sphericity).

In oder to investigate a potential carry-over effect, carry-over tests were calculated according to Wellek et al. [35].

Intra-individual pre-post differences for control sessions () and intervention sessions were calculated by substracting pre from post values (warm-up: measurement 2 –measurement 1; muscle fatigue: measurement 3 –measurement 2). For determining differences in the knee sounds between the sessions, repeated measures ANOVA was performed: once for the warm-up and once for the muscle fatigue effects. In addition, the estimation of effect size (η^2^) was calculated and interpreted according to Cohen [36]. The limits for the effect sizes were .01 (small effect), .06 (medium effect) and .14 (large effect). For checking the influence of the Tegner Activity Scale on the sound signal, the correlation with MSA or MDF was calculated.

## Results

Two participants had to be excluded from the analysis because of the occurrence of an exclusion criterion during the second measurement day (delayed onset muscle soreness) and missing data. No participant withdrew informed consent. Characteristics of the evaluated study population are summarized in Table 1.

**Table 1:** Characteristics of the evaluated study population.

Participants	N	age (years)	body mass (kg)	height (cm)	Tegner Activity Score
**Male**	7	26 ± 1.3	74.6 ± 13.4	180.76 ± 9.9	5.6 ± 1.7
		(23–30)	(63–86.8)	(174–190)	(4–9)
**Female**	10	25.5 ± 1.7	57.6 ± 5.6	168.4 ± 6.6	5.8 ± 1.3
		(23–28)	(45.2–68.4)	(160–180)	(4–8)
**Total**	17	25.7 ± 2	64.6 ± 11.6	173.9 ± 8.9	5.7 ± 1.6
		(23–30)	(45.2–86.8)	(160–190)	(4–9)

Data are displayed as means and standard deviations, in addition to the ranges (in brackets).

The mean, minimum and maximum values of the MSA and MDF at the medial tibia plateau and patella during the three sets of five repetitions for the three measurements (measurement 1, 2 and 3) in the intervention session and control session, separated for the two movement types of extension and flexion are depicted in Table 2. The time until exhaustion ranged from 2.8 to 15.2 minutes with an average of 8.3 minutes. The averaged Borg rating of perceived exertion after the warm-up was 7 (range 6–8) and, after muscle fatigue, this average was 16.5 (range 15–20).

**Table 2:** Mean (range) of the mean sound amplitude (A) and of the median power frequency (B) from the five repetitions of extension and flexion during the different measurement times in the control and intervention sessions.

**A**		**Measurement 1**		**Measurement 2**		**Measurement 3**	
		**Base control**	**Base inter-vention**	**Chair**	**Treadmill**	**Chair**	**Modified sit-to-stand**
**Medial tibia plateau**	**Extension**	88	88	86	89	86	88
		(78–99)	(80–96)	(69–98)	(84–98)	(70–97)	(82–97)
	**Flexion**	87	88	86	89	86	88
		(75–99)	(80–95)	(68–98)	(84–98)	(72–96)	(81–94)
**Patella**	**Extension**	97	94	95	90	95	89
		(83–104)	(71–103)	(80–104)	(75–103)	(80–104)	(67–101)
	**Flexion**	96	94	95	91	94	89
		(80–103)	(71–102)	(80–102)	(74–103)	(76–102)	(72–102)
**B**							
**Medial tibia plateau**	**Extension**	205	214	202	210	205	210
		(106–269)	(162–288)	(119–248)	(152–258)	(117–279)	(152–327)
	**Flexion**	197	225	201	211	204	211
		(117–276)	(152–323)	(126–298)	(161–266)	(126–295)	(138–310)
**Patella**	**Extension**	178	177	172	192	170	189
		(112–232)	(142–208)	(123–236)	(141–280)	(117–215)	(134–260)
	**Flexion**	178	193	186	198	182	197
		(125–244)	(145–231)	(132–237)	(135–253)	(129–239)	(148–250)

Measurement 1: baseline, Measurement 2: after 20 min sitting on a chair (Chair) or 20 min of treadmill walking (treadmill), Measurement 3: after 20 min sitting on a chair (Chair) or the modified sit-to-stand movement until task failure (modified sit-to-stand).

No carry-over effect was found for any parameter of the warm-up or muscle fatiguing exercise (p > .05).

### Effects of unspecific warm-up

**Fig 3:**
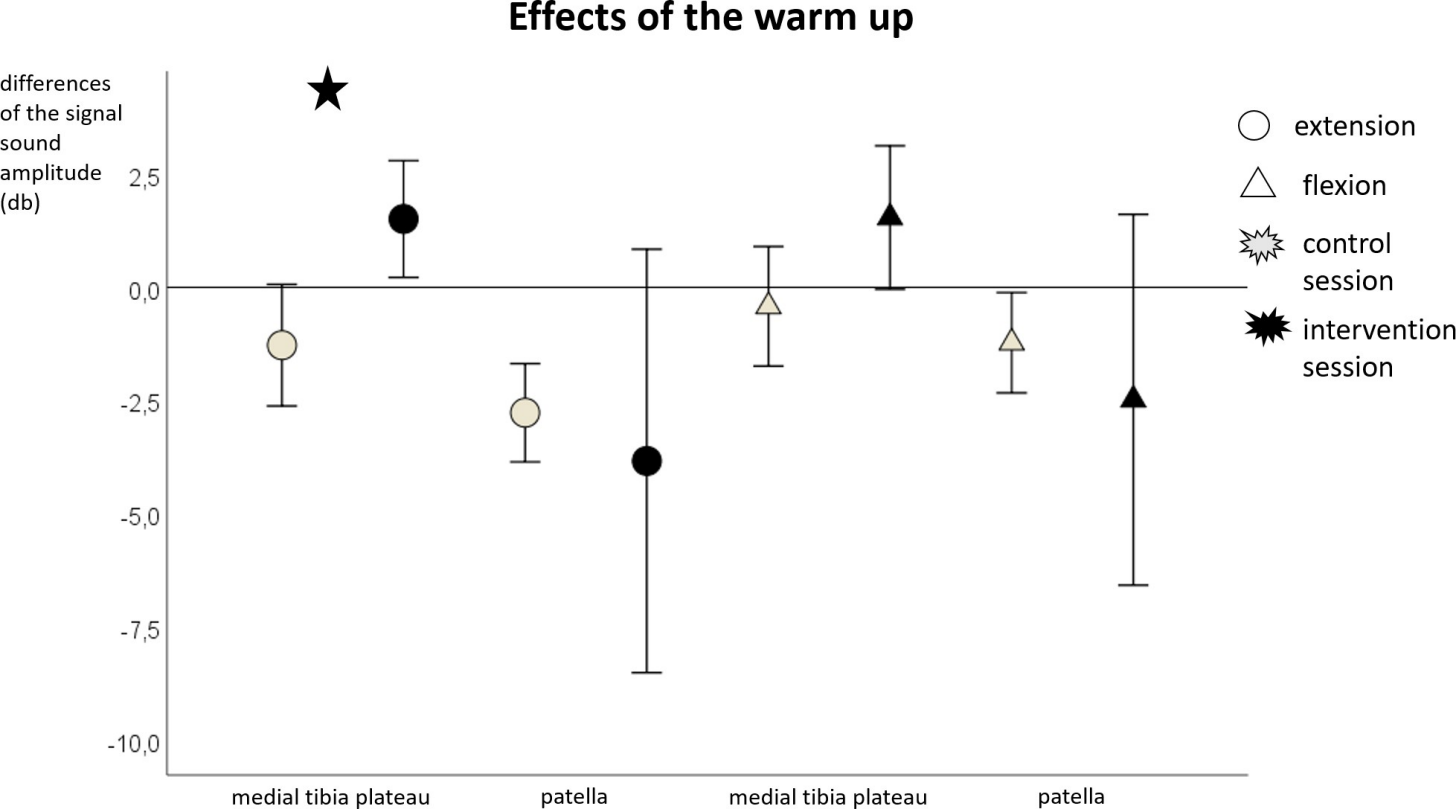
Mean and 95% confidence interval values of the pre-post differences of the mean sound amplitude during extension) and flexion) for the warm-up. The asterisks mark significant differences between the control and intervention sessions.

**Fig 4:**
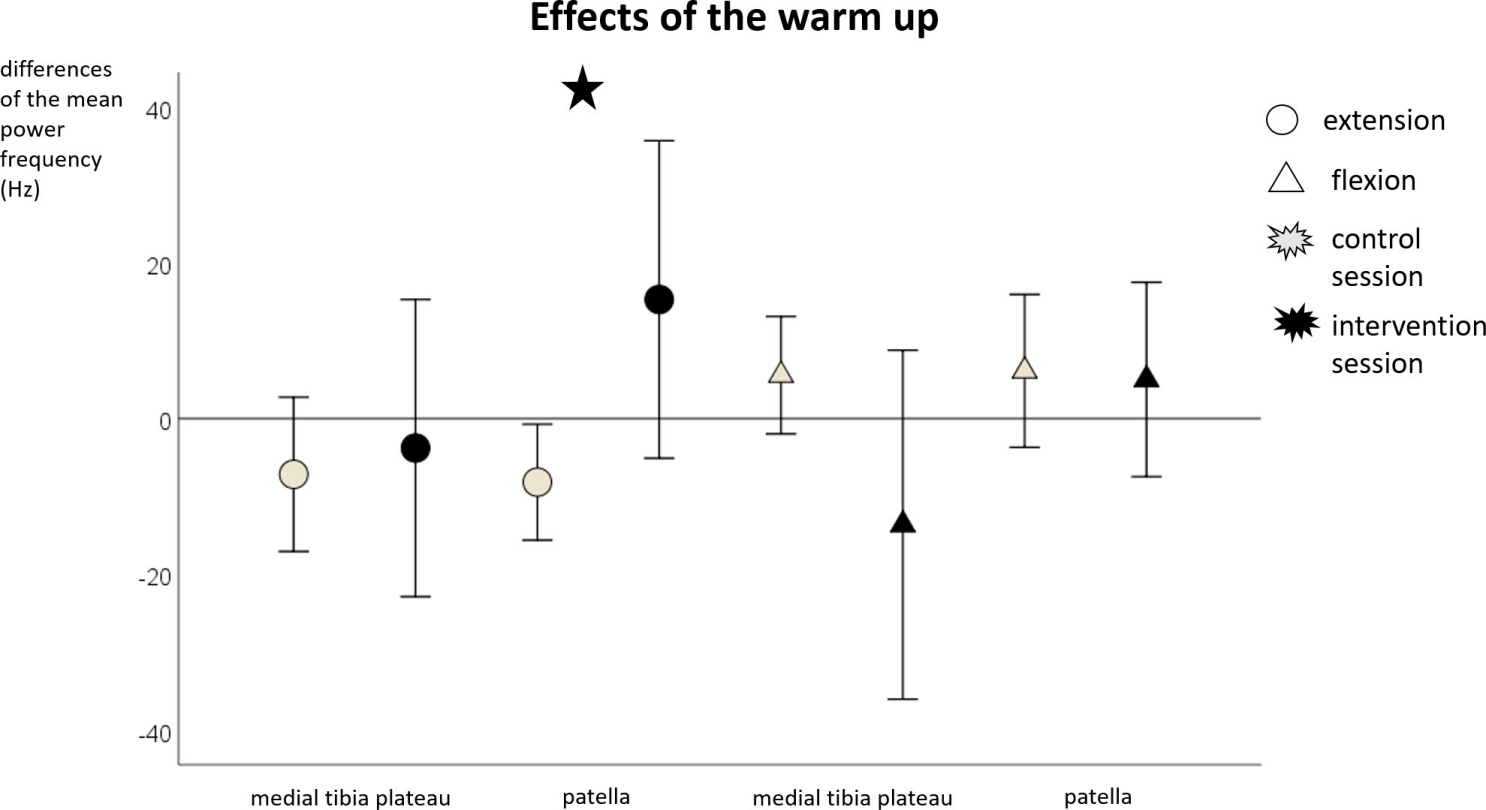
Mean and 95% confidence interval values of the pre-post differences of the median power frequency during extension and flexion for the warm-up. The asterisks marks significant differences between the control and intervention sessions.

We found a significant difference between the sessions for the MSA at the medial tibia plateau (F = 9.5; p = .007; η^2^ = .37) and for the MDF at the patella (F = 5.7; p = .03; η^2^ = .26) during extension. Furthermore, there was a tendency for a significant difference between the sessions for the MSA at the medial tibia plateau during flexion (F = 4.3; p = .055; η^2^ = .21). The median and 95% confidence interval values of the pre-post differences in the control and intervention session for the MSA and MDF can be seen in Figs 3 and 4.

### Effects of muscle fatiguing exercise

**Fig 5:**
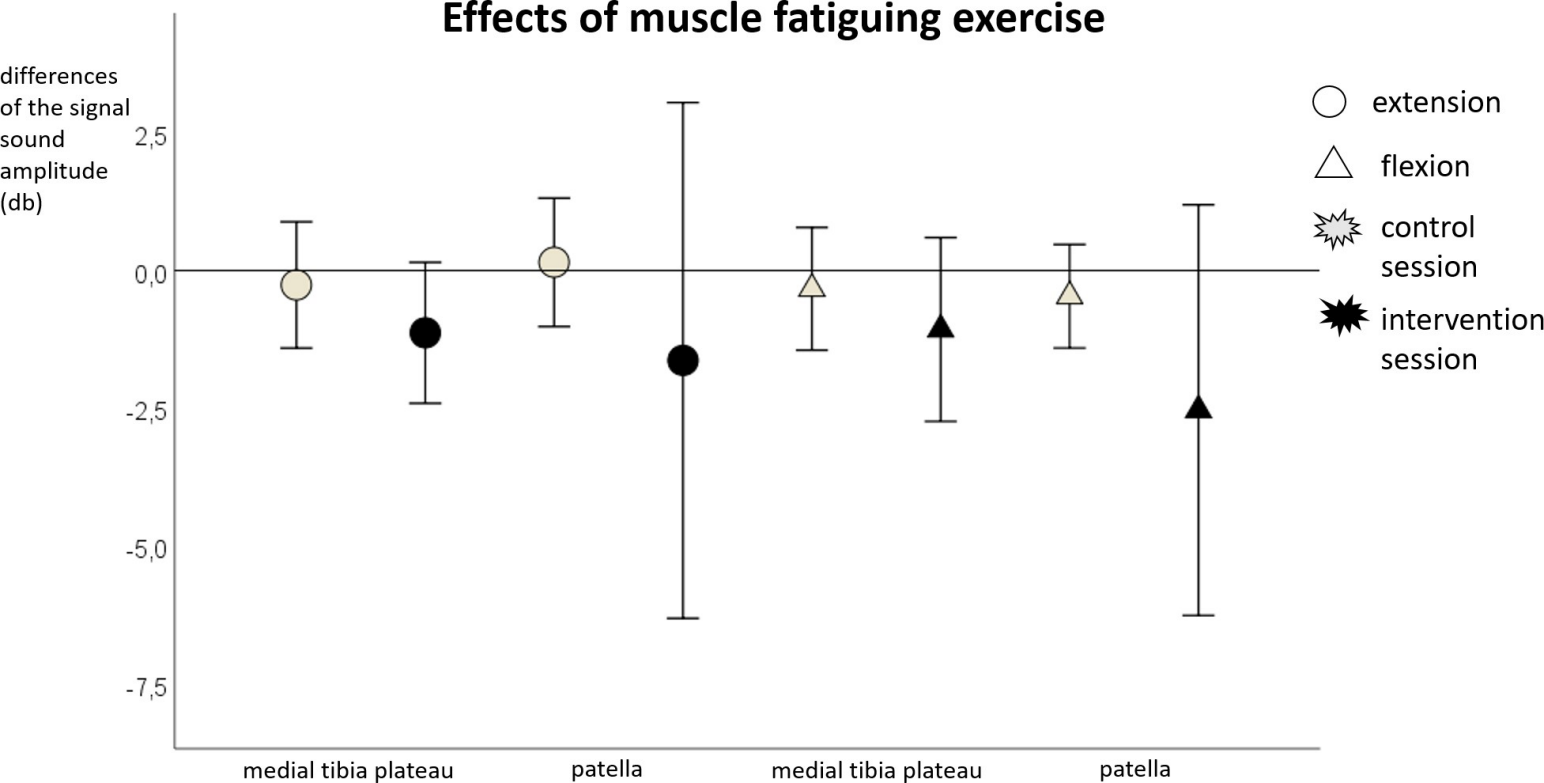
Mean and 95% confidence interval values of the pre-post differences of the mean sound amplitude during extension and flexion for muscle fatigue.

**Fig 6:**
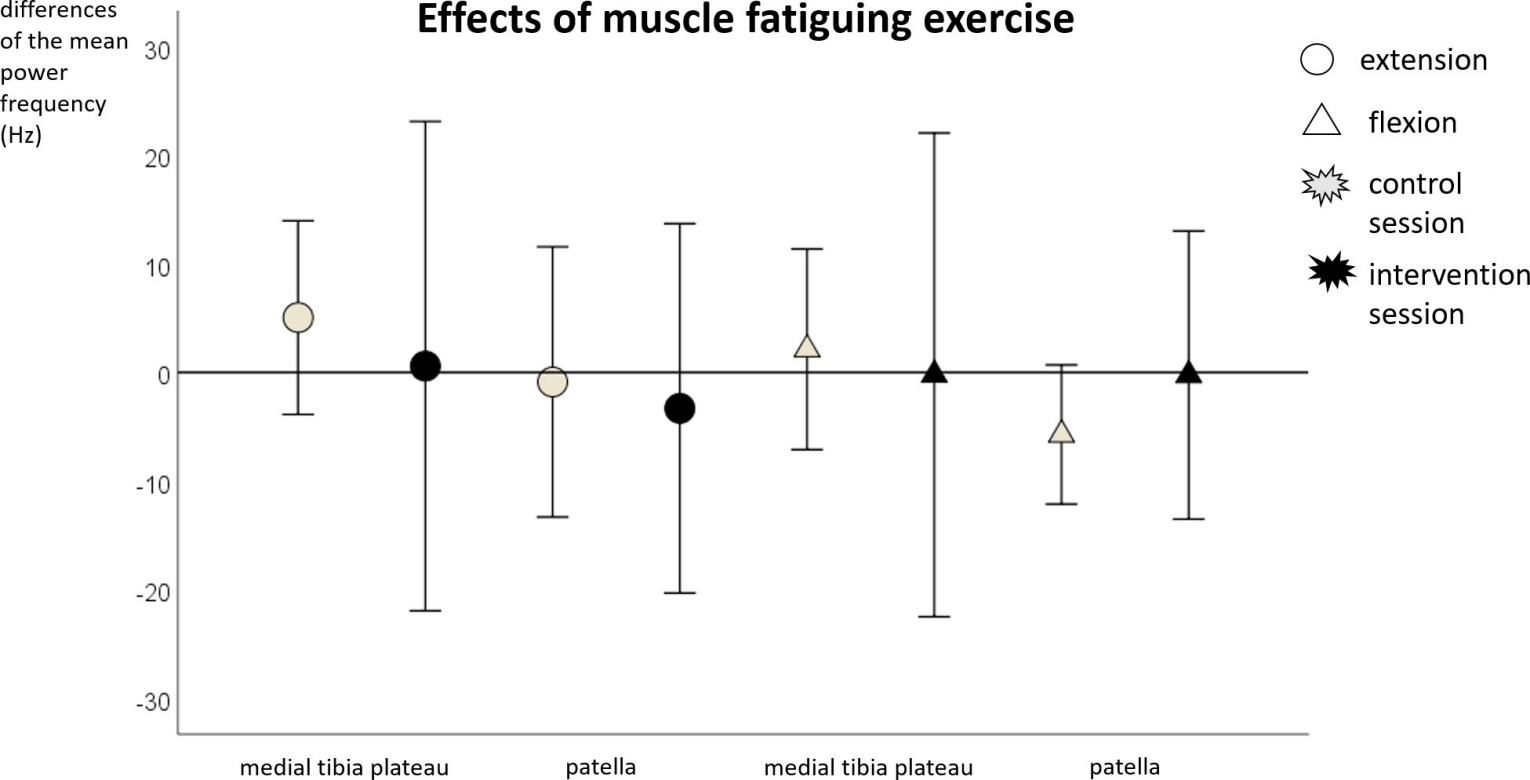
Mean and 95% confidence interval values of the pre-post differences of the median power frequency during extension and flexion for muscle fatigue.

There were no significant differences in the ANOVA for any of the parameters (p > .05). The median and 95% confidence interval values of the pre-post differences in the control and intervention sessions for the MSA and MDF can be seen in Figs 5 and 6.

### Explorative analyses

Significant correlations could be found between the Tegner Activity Scale and MSA at the patella during extension (r = -.52; p = .03), the Tegner Activity Scale and MSA at the patella during flexion (r = -.66 p = .004) as well as the Tegner Activity Scale and MDF at the patella during extension (r = .64; p = .006) after the warm-up.

## Discussion

With the aim to analyzes the potential effects of a warm-up and a fatiguing exercise on knee joint sounds, the hypotheses of a reduction in mean sound amplitude (MSA) after the warm-up and an increase in MSA after muscle fatigue could not be confirmed.

In contrast, we found a significant increase in knee joint sound MSA at the medial tibia plateau during extension and a tendency for a significant increase during flexion after the warm-up. No significant effects on the MSA at the patella (neither during extension nor during flexion) were found after the warm-up. The fatiguing protocol had no effect on any vibroarthrographic outcomes. Furthermore, a significant increase in median power frequency (MDF) at the patella during extension was found after the warm-up. No effect was found for the muscle fatiguing exercise.

### Effects of the warm-up

The increase in MSA at the medial tibia plateau during extension after the warm-up may be explained by increased knee load [4–6]: as the external load was the same, the internal knee load could be increased. Walking on the treadmill may have caused the fluid to flow out of the extracellular matrix of the cartilage, resulting in an increased internal load on the joint [37]. In addition, increased load on the knee leads to stiffening of the cartilage [38]. Furthermore, a study on cartilage deformation showed a 2.8% cartilage decrease in the knee joint after 5 minutes of normal walking on level ground [39]. These altered cartilage properties may have led to a poor arthrokinematic of the joint and consequently increased kinetic friction resulting in a cumulative load increasing effect of the warm-up. up.

However, a study that compared vibroarthrography [34] with electromyography showed that the signal amplitude of the two methods increase simultaneously [40]. In this respect, increased activation of the muscles originating from or attaching to the medial tibia tuberosity (e.g., musculus sartorius, gracilis, semimembranosus, semitendinosus, vastus medialis) may have influenced the MSA [41]. In addition, it is conceivable that an accelerated muscle activation may have increased knee moments [28] and thus MSA [7]. From this perspective, the increased MSA may also reflect higher muscle activity.

The increase in MDF found at the patella can not be attributed explicitly to knee load [9, 10, 28]. On the one hand, Bączkowicz et al. [5] established the connection between the knee joint sounds and the arthrokinematic motion quality and showed that an unloaded flexion-extension movement in healthy participants is almost vibration-free, whereas standing up and sitting down is associated with higher sound amplitude and frequency. Thus, it appears that the arthrokinematic movement quality may be of greater relevance in closed chain movements which can be based on the increased joint compressive forces [42]. It is possible that the warm-up may have increased the contact stress of the knee joint resulting in a poorer arthrokinematic motion quality [5], which may be due to a decrease in cartilage volume after walking on the treadmill. However, most authors agree, that the sound frequency does not depend primarily on the physiological changes of the joint, but more on external factors such as the type of movement [4, 7, 8]. Since the movement type has not changed in the present study, it is also conceivable that the warm-up has led to biomechanical or neuromuscular changes.

It is conspicuous that significant differences were only found during extension, this may be related to the higher muscle activity [43] and knee loading [28] during standing up in comparison to sitting down. However, there was also a tendency for a significant increase in MSA at the medial tibia plateau during flexion. Furthermore, the large standard deviations of the MSA at the patella (Figs 3–6*)* are conspicuous. Since the data was checked for artifacts, it appears that the participants showed interindividual responses, especially at the patella. In addition, particularly the knee sound signal at the patella correlated with the Tegner Activity Scale so that the activity level of the participants could have led to the interindividual responses.

In summary, the increased MSA and MDF could indicate a higher load on the knee joint with poorer arthrokinematic motion quality due to cartilage deformation [39] or higher compressive forces on the joint [5] The results indicate that walking on the treadmill may not be considered as a joint-gentle warm-up.

### Effect of muscle fatigue

We suspected that a muscle fatiguing exercise until task failure would result in an increase of knee joint sound due to the high load on the knee joint (especially the high contact pressure at the knee during the modified sit-to-stand, [44]) and loss of neuromuscular control [20–24]. Contrary to our assumption, the muscle fatiguing exercise appears to have no effect on knee sounds during sit-to-stand in a healthy, active group of participants with non-symptomatic knees. The study from Kalo et al. [6] has already ascertained that knee sound increases with an increase in load, however, in the present study, the knee sounds were not measured during the knee loading task, but after task failure, which could mean that the effects of the load do not persist and do not play a role during an activity of daily living like sit-to-stand. According to Enoka and Duchateau [45], muscle fatigue can be caused by different mechanisms, e.g., by an accumulation of metabolites in the muscle or loss of motor control and it is typically defined as a decrease in maximal performance. However, this means that submaximal performances, such as an activity of daily living, are not necessarily affected. In addition, the studied collective included athletic and young participants. A bilateral lower limb fatigue protocol of Longpré et al. [46] did not change the knee moment, angle, stiffness or muscle co-activation of young and healthy women; only the peak knee extension moment was found to be reduced after the fatiguing protocol.

Furthermore, in a standardized fatigue protocol, the fatigue reactions of the participants can be very individual [47]. The fatigue protocol used in the present study may have caused facilitating reactions in some participants [48], as the time until muscle fatigue varied greatly and may have been too short for some participants. It is probable that the MDF could be remedied by prolonged fatigue [49], thus, it is also possible that the load on the knee joint was not great or applied long enough to sustain local effects in the knee joint (e.g., an increase in the viscosity of the synovial fluid).

### Limitations and future perspective

A limitation of the present study is the limited number of participants, which is why this is merely a pilot study. Many more participants are needed in future research. In addition, the present study did not measure muscle activity, knee joint moments or knee temperature. These measurements should be integrated into future studies in order to draw conclusions about any underlying mechanisms of joint stabilization, load and lubrication. Furthermore, the data were collected in asymptomatic, young participants and, thus, cannot be applied to older people or patients. As this study has shown that knee sounds can be influenced during the conduction of the same exercise by, for example, preliminary movements, it would be of relevance to integrate a standardized warm-up protocol for the measurement of knee joint sounds. In addition, the effect of a sport-specific or joint-gentle warm-up on knee joint sounds should be investigated.

## Conclusion

The present study investigated, for the first time, the changes in knee sounds after a warm-up and a muscle fatiguing exercise in a healthy and active study population with non-symptomatic knees during the sit-to-stand movement. We demonstrated a significant increase in MSA at the medial tibia plateau and a significant increase in MDF at the patella during extension (from sit-to-stand), as well as a tendency for a significant increase in MSA at the medial tibia plateau during flexion (from stand-to-sit) after walking on a treadmill. However, no impact of the muscle fatiguing exercise on knee joint sounds during the activity of daily living was found.

Further studies using a larger study sample are needed to confirm our results on the effect of a warm-up on knee joint sounds. Moreover, these studies should integrate physiological and kinetic measurements to examine the underlying mechanisms. In addition, a joint-gentle standardized warm-up for measuring knee joint sounds should be investigated.

## Supporting information

S1 Table Data table with the mean and median values from five repetitions of extension and of the mean sound amplitude (A) and of the median power frequency (B) during the different measurement times in the control and intervention sessions, as well as the pre-post differences for the corresponding conditions.
(XLSX)
